# Machine Learning Predictive Modeling for the Identification of Moderate Coronavirus Disease 2019 During the Pandemic: A Retrospective Study

**DOI:** 10.7759/cureus.50619

**Published:** 2023-12-16

**Authors:** Tao Wang, Zhanqing Zhao, Wenzhe Li, Jing Wu, Qianru Ye, Hui Xie

**Affiliations:** 1 Department of Critical Care Medicine, Shanghai General Hospital, Shanghai, CHN; 2 Department of Critical Care Medicine, Hainan Western Central Hospital, Danzhou, CHN; 3 Department of Critical Care Medicine, Ruijin Hospital, Shanghai Jiao Tong University School of Medicine, Shanghai, CHN

**Keywords:** pandemic, retrospective study, predictive model, moderate covid-19, machine learning

## Abstract

Background: Timely differentiation of moderate COVID-19 cases from mild cases is beneficial for early treatment and saves medical resources during the pandemic. We attempted to construct a model to predict the occurrence of moderate COVID-19 through a retrospective study.

Methods: In this retrospective study, clinical data from patients with COVID-19 admitted to Hainan Western Central Hospital in Danzhou, China, between August 1, 2022, and August 31, 2022, was collected, including sex, age, signs on admission, comorbidities, imaging data, post-admission treatment, length of stay, and the results of laboratory tests on admission. The patients were classified into a mild-to-moderate-type group according to WHO guidance. Factors that differed between groups were included in machine learning models such as Bernoulli Naïve Bayes (BNB), linear discriminant analysis, support vector machine (SVM), least absolute shrinkage and selection operator (LASSO), and logistic regression (LR) models. These models were compared to select the optimal model with the best predictive efficacy for moderate COVID-19. The predictive performance of the models was assessed using the area under the curve (AUC), sensitivity, specificity, and calibration plot.

Results: A total of 231 patients with COVID-19 were included in this retrospective analysis. Among them, 152 (68.83%) were mild types, 72 (31.17%) were moderate types, and there were no patients with severe or critical types. A logistic regression model combined with age, respiratory rate (RR), lactate dehydrogenase (LDH), D-dimer, and albumin was selected to predict the occurrence of moderate COVID-19. The receiver operating characteristic curve (ROC) showed that AUC, sensitivity, and specificity in the model were 0.719, 0.681, and 0.635, respectively, in predicting moderate COVID-19. Calibration curve analysis revealed that the predicted probability of the model was in good agreement with the true probability. Stratified analysis showed better predictive efficacy after modeling for people aged ≤66 years (AUC = 0.7656) and a better calibration curve.

Conclusion: The LR model, combined with age, RR, D-dimer, LDH, and albumin, can predict the occurrence of moderate COVID-19 well, especially for patients aged ≤66 years.

## Introduction

It has been nearly three years since the outbreak of COVID-19, which infected more than 600 million people and killed more than six million [1] worldwide. The transmission of the SARS-Co-2 virus has been increasing due to continuous mutations, such as Omicron variants, although their virulence has decreased [2]. Most mild cases present mild upper respiratory symptoms such as nasopharyngeal discomfort and cough [3]. Although most cases are asymptomatic or mildly symptomatic, there is still a proportion of patients with significant lung damage and even multiple organ dysfunction who require hospitalization [4]. To improve the prognosis of these patients, timely screening and treatment are particularly important. Radiographic imaging, such as CT scans and X-rays, plays an important role in the screening of these patients, according to WHO guidelines [5]. However, the imaging equipment may not be available due to limited medical resources during the pandemic. Therefore, it is important to screen patients using other approaches. We attempted to develop machine learning models to predict the occurrence of lung injury by retrospectively analyzing existing clinical cases.

## Materials and methods

Study design and patients

A retrospective analysis of clinical data from adult patients with COVID-19 who were admitted to Hainan West Central Hospital in Danzhou, China, from August 2022 and September 2022 was performed. The data were collected from the electronic medical record system of Hainan West Hospital. Data included sex, age, vital signs on admission, comorbidities, imaging data, treatment, length of stay, and results of the first laboratory test after admission. Mild COVID-19 and moderate COVID-19 were diagnosed according to WHO guidelines [5]. Mild COVID-19 was defined as symptomatic patients meeting the case definition for COVID-19 without evidence of viral pneumonia or hypoxia. Moderate COVID-19 was defined as patients with clinical signs of pneumonia (fever, cough, dyspnea, and fast breathing) but no signs of severe pneumonia, including oxygen saturation (SpO_2_) ≥ 90% on room air. A total of 231 patients with confirmed COVID-19 were included in the study. All patients had coronavirus polymerase chain reaction (PCR) tests confirming the Omicron variant (BA.5.1.3).

Data collection

Demographic characteristics of the patients, vital signs on admission, comorbidities, imaging data, disease type, vaccination status prior to onset, treatment after admission, length of stay, and results of the first laboratory tests after admission were obtained through the electronic medical record system. Demographic characteristics included age, sex, height, and weight; vital signs at admission included blood pressure, heart rate, respiratory rate, and temperature; and underlying diseases included diabetes, cardiovascular disease, cerebrovascular disease, chronic lung disease, chronic liver disease, chronic kidney disease, solid tumors, hematologic diseases, and immunodeficiency diseases. Laboratory tests included routine blood tests, biochemistry, electrolytes, C-reactive protein (CRP), procalcitonin (PCT), coagulation tests, and D-dimer.

Statistical analysis

Data processing and analysis were performed using the Smart Research Online platform (https://dxonline.deepwise.com/). Patients were grouped according to the COVID-19 severity classification. Categorical variables were compared using the chi-square test or Fisher's exact test and expressed as n (frequency). Continuous variables with a normal distribution were compared using a t-test and expressed as the mean ± standard deviation. Continuous variables with non-normal distributions were compared using the Mann-Whitney U test and are represented by the median and interquartile range (IQR). Spearman’s correlation coefficient was used for correlation analysis. Correlations between factor variables that were significantly different between mild and common types were analyzed. P-values <0.05 were considered to be statistically significant. Variables that differed between groups were included in machine learning models, and predictive modeling was performed using machine learning models including plain Bayesian, linear discriminant analysis, support vector machine (SVM), and least absolute shrinkage and selection operator (LASSO), and logistic regression (LR) models, and the predictive efficacy of these models was compared.

The predictive efficacy of these models was evaluated by receiver operating characteristic (ROC) curves, and the optimal model was selected by sensitivity, specificity, and area under the curve (AUC). The AUC was calculated, and an AUC of >0.7 was considered to be a good model. The calibration curve was used to assess the agreement between the predicted probabilities of the model and the actual probabilities.

## Results

Clinical and laboratory characteristics of COVID-19 patients on admission

A total of 231 COVID-19 patients were included in this retrospective analysis. Among them, 152 (68.83%) were mild cases, 72 (31.17%) were moderate cases, and there were no severe or critical cases. On admission, there were no statistically significant differences between the two groups in terms of sex, weight, height, body temperature, systolic blood pressure (SBP), heart rate, days of positive nucleic acid testing, vaccination status, or underlying disease status (including diabetes mellitus, cardiovascular disease, cerebrovascular disease, chronic lung disease, chronic liver disease, chronic kidney disease, and solid tumor) (P >0.05). No significant difference was found between the two groups in the laboratory tests, including for white blood cell count (WBC), lymphocyte count, hemoglobin level, platelet count, CRP, aspartate aminotransferase (AST), prealbumin, bilirubin level, and blood creatinine levels (P >0.05). Patients in the moderate group were significantly and statistically older than those in the mild group (years, 45.50 (31.25-61.50) versus 61.50 (52.00-70.00, P <0.05). Diastolic blood pressure (DBP)(mmHg, 72.72±10.23 versus 76.68±9.52), respiratory rate (breaths/min, 19.46±1.65 versus 20.21±1.53), PCT (ng/mL, 0.02 (0.01-0.04) versus 0.02 (0.01-0.05)), interleukin 6 (IL6) levels (pg/ml, 10.80 (6.25-17.70) vs. 13.80 (9.50-24.50)), lactate dehydrogenase(LDH) levels (U/L, 214.87±45.39 vs. 229.15±48.92), glutamate transaminase (U/L,18.00 (13.00-28.00) vs. 21.00 (16.00-30.00)), and urea nitrogen (mmol/L,3.70 (2.90-4.60) versus 4.15 (3.33-5.13)) were higher than in the moderate group (P <0.05). However, the albumin level was significantly lower in the moderate group (g/L, 41.26±4.44 versus 39.06±4.26, P = 0.001) (Table 1).

**Table 1:** Clinical characteristics of patients with mild and moderate COVID-19 on admission COVID-19: coronavirus disease 2019; RR: respiratory rate; IQR: interquartile range; SBP: systolic blood pressure; DBP: diastolic blood pressure; PCR: polymerase chain reaction; WBC: white blood cell count; CRP: C-reactive protein; PCT: procalcitonin; ALT: alanine aminotransferase; AST: aspartate aminotransferase; IL6: interleukin 6; LDH: lactate dehydrogenase *P-values <0.05 were considered statistically significant.

Characteristics	Mild COVID-19 (N=159)	Moderate COVID-19 (N=72)	P-value
Sex, n (%)	-	-	0.053
Female	101 (63.5%)	36 (50.0%)	
Male	58 (36.5%)	36 (50.0%)	
Age (years, IQR）	45.50 (31.250-61.50)	61.50(52.000-70.00)	p<0.001*
Height (cm, IQR）	161.00(158.00-169.00)	165.00(153.00-170.00)	0.867
Body temperature (℃,mean ± SD）	36.95±0.72	37.12±0.79	0.235
RR (mean ± SD)	19.46±1.65	20.21±1.53	0.019 *
Pulse rate (bpm, IQR）	80.00 (78.00-85.00)	80.00(78.00-89. 00)	0.296
SBP (mmHg, mean ± SD）	124.09±16.51	126.29±11.11	0.458
DBP (mmHg, mean ± SD）	72.72±10.23	76.68±9.52	0.047 *
PCR positive days before admission (days, IQR）	1.00 (1.00-3.00)	1.000(1.00-3.75)	0.345
Vaccination history	-	-	0.396
No vaccination history, n (%)	2 (2.7%)	1 (3.3%)	
Vaccinated once, n (%)	1 (1.4%)	2 (6.7%)	
Vaccinated twice, n (%)	25 (34.2%)	7 (23.3%	
Vaccinated three times, n (%)	45 (61.6%)	20 (66.7%)	
Diabetes mellitus, n (%)	8 (5.0%)	4 (5.6%)	1
Cardiovascular disease, n (%)	17 (10.7%)	12 (16.7%)	0.204
Cerebrovascular disease, n (%)	5 (3.1%)	3 (4.2%)	0.996
Chronic lung disease, n (%)	7 (4.4%)	8 (11.1%)	0.103
Chronic liver disease, n (%)	5 (3.1%)	5 (6.9%)	0.334
Chronic kidney disease, n (%)	2 (1.3%)	2 (2.8%)	0.783
Solid tumor, n (%)	2 (1.3%)	3 (4.2%)	0.358
Complicated acute respiratory failure, n (%)	0 (0.0%)	2 (2.8%)	0.179
Complicated acute liver function impairment, n (%)	0 (0.0%)	2 (2.8%)	0.179
Progress to severe COVID-19, n (%)	0 (0.0%)	2 (2.8%)	0.179
WBC（109/L, mean ± SD）	5.19±2.05	5.54±2.28	0.268
Lymphocyte count (109/L, mean ± SD)	1.25±0.68	1.19±0.70	0.567
PLT（109/L, mean ± SD）	204.09±65.22	204.21±77.24	0.991
Hb（g/L, IQR）	128.00 (118.00-139.00)	124.00 (114.00-141.50)	0.357
CRP (mg/L, IQR)	4.49 (2.06-9.23)	5.46 (2.03-14.58)	0.19
PCT（ng/mL ,IQR)	0.02 (0.01-0.04)	0.02 (0.01-0.05)	0.040
IL-6 (pg/ml, IQR)	10.80 (6.25-17.70)	13.80 (9.50-24.50)	0.012 *
D-dimer（ng/ml, IQR）	460.00 (240.00-740.00)	630.00 (280.00-1015.00)	0.032 *
ALT（u/L, IQR）	18.00 (13.00-28.00)	21.000 (16.00-30.00)	0.021 *
AST（u/L, IQR）	34.00 (26.00-45.00)	37.00 (30.00-50.50)	0.061
Prealbumin（g/L, IQR）	191.00 (167.00-225.00)	196.00 (156.25-237.75)	0.994
Albumin (g/L, mean ± SD)	41.26±4.44	39.06±4.26	0.001 *
LDH (U/L,mean ± SD)	214.87±45.39	229.15±48.92	0.045 *
Total bilirubin (umol/L, IQR）	8.70 (6.80-11.80)	10.50 (7.05-13.98)	0.153
Direct bilirubin (umol/L, IQR）	1.90 (1.40-2.50)	1.95 (1.60-2.68)	0.214
Blood urea nitrogen (mmol/L, IQR）	3.70 (2.90-4.60)	4.150 (3.33-5.13)	0.021 *
Serum creatinine (umol/L, IQR）	67.00 (57.00-85.00)	71.00 (56.25-82.50)	0.598

Developing a model to predict the occurrence of moderate COVID-19

**Figure 1:**
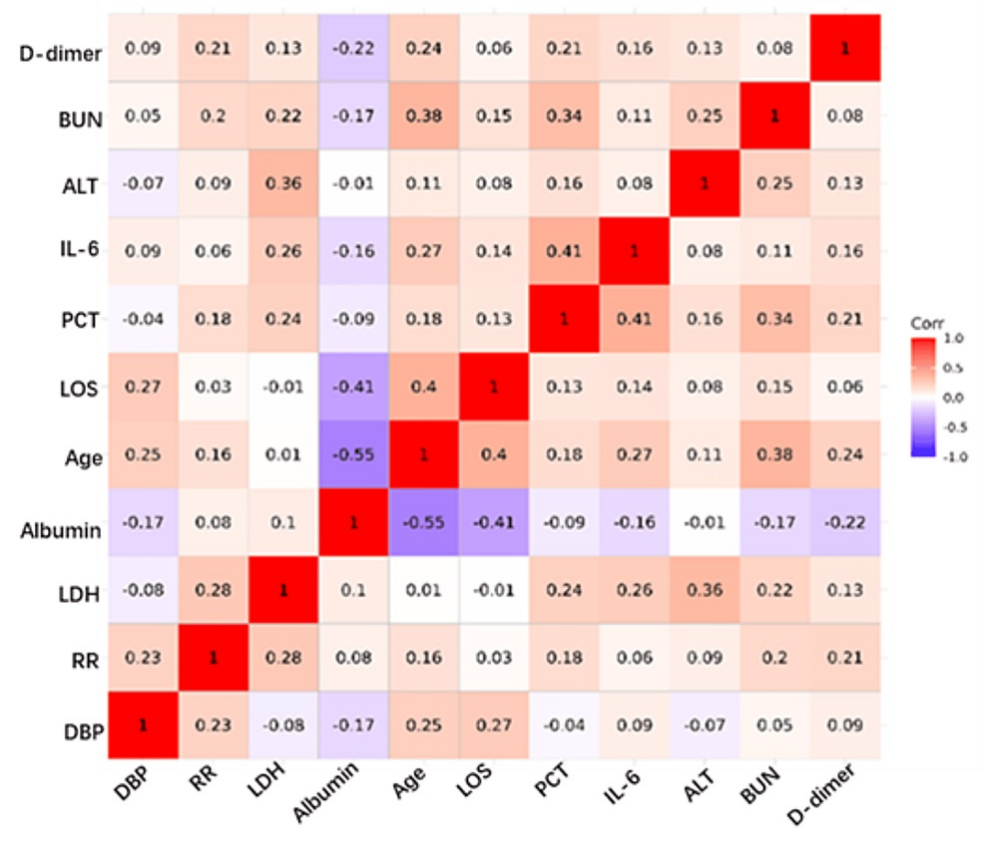
Spearman's analysis showed the correlation of 11 significantly different variables. DBP: diastolic blood pressure; RR: respiratory rate; LDH: lactate dehydrogenase; LOS: length of stay; PCT: procalcitonin; AST: aspartate aminotransferase; IL6: interleukin 6; ALT: alanine aminotransferase; BUN: blood urea nitrogen

Spearman's analysis showed that the correlation between all of the above variance variables was low (Figure 1), so these variables could be included in the analysis model.

The variance factors between the two groups were included in the Bernoulli Naïve Bayes (BNB), linear discriminant analysis, SVM, and LR models. By comparing the indicators between the models, we found that the LR model had the best sensitivity (sensitivity = 0.653) and Youden's index = 0.288 (Table 2), so the LR model was selected for modeling.

**Table 2:** Predictive efficacy of BNB, linear discriminant analysis, SVM, and logistic regression models for moderate COVID-19. AUC: area under the curve; BNB: Bernoulli Naïve Bayes; SVM: support vector machine; LR: logistic regression

Models	AUC	Sensitivity	Specificity	Youden's Index
BNB	0.710	0.347	0.799	0.146
Linear discriminant analysis	0.707	0.222	0.893	0.115
SVM	0.704	0.083	0.943	0.027
LR	0.705	0.653	0.635	0.288

**Figure 2:**
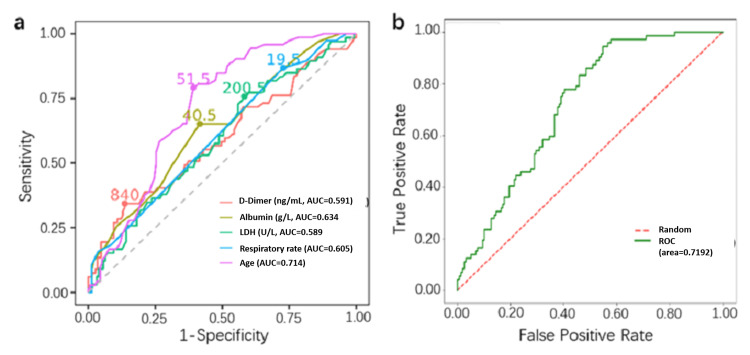
a. Receiver operating characteristic curve of age, D-dimer, LDH, respiratory rate, and albumin; b. Receiver operating characteristic curve of the LR model. LDH: lactate dehydrogenase; LR: logistic regression

The top five variables on the obtained model feature weights were selected as age, D-dimer, LDH, respiratory rate, and albumin (Table 3), and their AUC values for the prediction of moderate COVID-19 were 0.714, 0.591, 0.589, 0.605, and 0.634 (Figure 2a).

**Table 3:** Variables and characteristic coefficient, relative weights in the logistic regression model. LDh: lactate dehydrogenase; LR: logistic regression

Characteristics	Characteristic coefficient	Relative weight
Age	0.718	1
D-dimer	0.300	0.418
LDH	0.279	0.388
RR	0.266	0.371
Albumin	-0.210	-0.293

When these five variables were incorporated into the final LR model, the AUC, sensitivity, and specificity were 0.719, 0.681, and 0.635, respectively, for predicting the occurrence of moderate COVID-19 (Table 4, Figure 2b).

**Table 4:** Predictive efficacy of moderate COVID-19 after modeling with a logistic regression model. AUC: area under the curve

Model	AUC	Sensitivity	Specificity
Logistic regression	0.719	0.681	0.635

**Figure 3:**
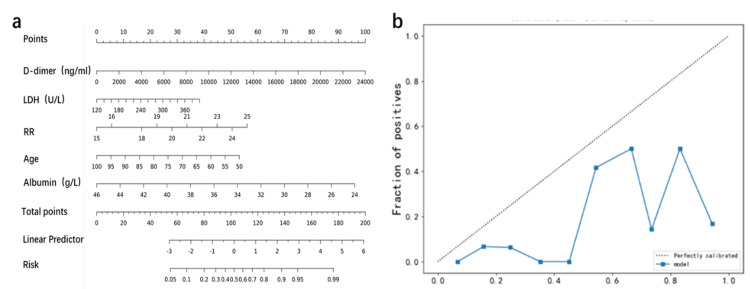
(a) A nomogram illustrating the scores for age, D-dimer, LDH, respiratory rate, and albumin in predicting moderate COVID-19. (b) Calibration curve. LDH: lactate dehydrogenase; RR: respiratory rate

To facilitate clinical application, the LR model was visualized using the nomogram. Scores were assigned to each variable of the model, and the scores were summed to calculate the total score to reflect the probability of moderate COVID-19 for each patient (Figure 3a).

We found good agreement between the predictive probability of the model (predicted values) and the true probability (observed values) by calibration curve analysis (Figure 3b).

We stratified the patients by age to assess the predictive efficacy of the model across age groups. After grouping by age in quartiles (<35 years, 35-52 years, 53-66 years, >66), we found that the predictive efficacy of our LR model was better in the first three-quarters of the age quartile (≤ 66 years, AUC = 0.766, Table 5).

**Table 5:** Predictive efficacy of the logistic regression model for moderate COVID-19 in patients aged ≤66. AUC: area under the curve

Model	AUC	Sensitivity	Specificity
Logistic regression	0.766	0.714	0.615

**Figure 4:**
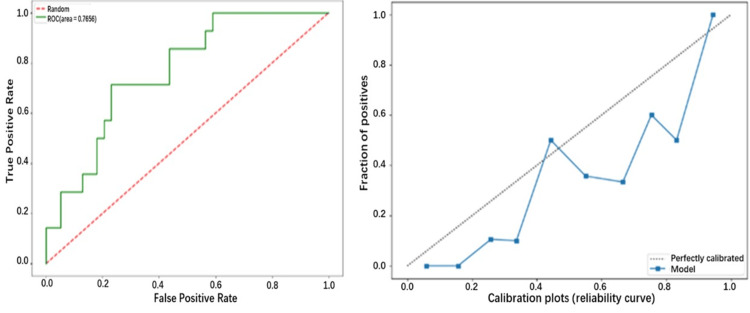
(a) Receiver operating characteristic curve of the logistic regression model in patients ≤66 years (b) Calibration curve

The calibration curve showed better agreement between the predicted values and the observed values in the model (Figures 4a-4b).

Model applications

We used nomograms and online links to assist clinicians in performing rapid screening. The LR model was visualized and applied using a nomogram. The model assigned a score for each variable, and the scores were summed up to calculate a total score reflecting the probability of moderate COVID-19 for each patient. To obtain the patient's outcome and the corresponding probabilities to assist in clinical applications, an online link was generated (https://dxonline.deepwise.com/prediction/index.html?baseUrl=%2Fapi%2F&id=19350&topicName=undefined&from=share&platformType=wisdom).

## Discussion

Due to the general vulnerability of the population to SARS-CoV-2 and multiple routes of transmission [6], the pandemic has not yet ended. Although vaccines can partially stop transmission [7], the virus can achieve immune evasion through continuous mutation [8], and some variants increase the transmissibility of the virus, especially the currently prevalent Omicron variant. However, due to differences in age, physiological status, immune status, and many other factors, patients present different manifestations when infected [9]. For example, most Omicron infections present with mild COVID-19 and only a small proportion of patients present with moderate or severe COVID-19. Identifying mild and moderate cases and implementing stratified management can save medical resources to a greater extent while enabling earlier treatment of moderate-type patients. Therefore, we attempted to analyze the clinical data of 231 patients with Omicron variant infection (both mild and moderate types) to identify the variables that differ between mild and moderate types, and based on the results, we have established a prediction model for moderate COVID-19.

By comparing mild and moderate cases, we found differences in age, respiratory rate, D-dimer, LDH, and albumin between the two groups, suggesting that old age and hypoproteinemia may be risk factors for progression to moderate COVID-19, and elevated respiratory rate, D-dimer, LDH, AST, urinary creatinine, PCT, and IL6 may indicate the development of moderate COVID-19. Incorporating the above factors into LR modeling revealed that age, D-dimer, LDH, respiratory rate, and albumin had the highest characteristic weights in the model. The occurrence of moderate pneumonia was well predicted after modeling using the five variables mentioned above. After modeling patients aged ≤66 years, we found that age, D-dimer, LDH, respiratory rate, and albumin still had the highest characteristic weights in the model, and the model had better efficacy in predicting moderate COVID-19 with higher AUC values.

We found that age had the highest weight value in the above model, with an AUC of 0.714 in the univariate prediction model, suggesting that old age was an important risk factor for progression to the moderate type in patients with mild disease. Early in the epidemic, Wu et al. found that older patients were more likely to develop acute respiratory distress syndrome (ARDS) [10]. An analysis of COVID-19 patients in 45 countries by O'Driscoll et al. found that these patients showed significant age-specific outcomes, with a log-linear increase by age among individuals older than 30 years [11]. According to the Centers for Disease Control and Prevention (CDC), the mortality rate for people aged >75 years is more than 100 times that for those aged 18-29 years [12]. This may be related to decreased immune function in elderly patients. With increasing age, the migration, differentiation, and cytokine production of innate immune cells are impaired or delayed, while adaptive immune B- and T-cell functions deteriorate [13], and the immune system's ability to resist viral replication and transmission decreases compared to that of younger patients. These changes may result in a significant increase in peak virus load [14], making elderly patients more vulnerable to lung and other organ involvement.

By comparing mild and moderate COVID-19, we found that the D-dimer level was higher in moderate COVID-19. Elevated D-dimer levels are an important indicator in response to coagulation disorders, and in our study, D-dimer levels were found to be higher in moderate COVID-19 patients than in patients with mild COVID-19. Previous studies have found elevations in approximately 36% of patients with COVID-19 [15], and elevated levels are correlated with higher ARDS risk, disease severity, and mortality [16, 17]. Coagulation disorders such as elevated dimers are associated with direct damage to multiorgan endothelial cells by the COVID-19 virus and the release of inflammatory factors such as IL6 caused by infection, leading to a hypercoagulable state, which can lead to an increased risk of thrombosis in the venous and arterial systems, as well as in the microvascular system of vital organs such as the lungs and kidneys [18]. An autopsy of patients who died of COVID-19 revealed the presence of diffuse thrombosis in capillaries within the lungs [19]. In COVID-19 patients with D-dimer elevation, anticoagulation therapy has been proven to improve the prognosis of these patients [20]. Therefore, D-dimer can be used as a good predictor of the severity of COVID-19 and to evaluate the effect of treatment.

In our study, LDH levels were higher among moderate COVID-19 cases. Lactate dehydrogenase is an intracellular enzyme that maintains normal energy metabolism in the body with several isoenzymes, mainly in the heart, liver, kidney, lung, and striated muscle, and in the lung, mainly LDH-3 [21]. In COVID-19, damage to the lungs leads to more LDH release into the blood, causing an increase in LDH levels. In addition, the severe inflammatory response after viral infection can also damage the liver, heart, and other organs [22], which exacerbates the elevation of LDH. Henry et al. [23] found that elevated LDH was associated with a six-fold increase in the odds of severe and a 16-fold increase in mortality among COVID-19 patients through a pooled analysis of 1206 cases. Therefore, LDH can be used as an indicator to assess the severity of COVID-19.

In many clinical settings, hypoproteinemia is associated with increased severity and mortality [24], which is consistent with our findings: moderate COVID-19 cases had lower albumin levels than the mild type of COVID-19. A previous study found that hypoproteinemia increased disease severity and mortality. The probability of a poor prognosis was 70% in patients with hypoalbuminemia, compared to 24% in patients with normal albumin levels [25]. The potential mechanism of hypoalbuminemia associated with COVID-19 cases was thought to be related to direct viral damage, capillary leakage, and high protein catabolism due to a high inflammatory response [26].

In our results, the respiratory rate was significantly higher in moderate COVID-19 cases than in mild COVID-19 cases. The increase in respiratory rate reflects the aggravation of COVID-19. In some lung disease assessment methods, such as CURB-65 (an acronym for confusion, uremia, respiratory rate, BP, age ≥ 65 years), the pneumonia severity index (PSI), and the ROX index (defined as the ratio of oxygen saturation as measured by pulse oximetry/FIO2 to respiratory rate) [27, 28], and some systemic infectious disease assessment methods, such as the Sequential Organ Failure Assessment (SOFA) and acute physiology and chronic health evaluation (APACHE) II [29, 30], respiratory rate was included as an important parameter, and an increase in respiratory rate may indicate the aggravation of pneumonia or systemic disease. Therefore, the inclusion of respiratory frequency in our model improved its accuracy.

During the pandemic, it is particularly important to optimize the allocation of medical resources to treat focus groups due to the shortage of resources. Only symptomatic treatment is required for mild COVID-19, while further treatment and monitoring are required for moderate COVID-19. Our model could distinguish moderate COVID-19 from mild cases. Furthermore, we developed nomograms to make it easy for physicians to identify moderate cases so that they can receive treatment and monitoring earlier during the pandemic.

In this study, we created a model to predict the occurrence of moderate COVID-19 from clinical data and verified the good predictive efficacy of the model. However, our study also had some limitations. First, the small sample size had an impact on the predictive efficacy of the model; second, the prediction model was internally validated, which placed limitations on the evaluation of model efficacy and required further external validation; third, our model had not yet addressed the prediction of prognosis such as mortality. In addition, our model was built based on a population infected by the Omicron variant, and the prediction performance for other variants needs further validation.

## Conclusions

In this study, we developed a logistic regression model to predict the occurrence of moderate COVID-19 and evaluated its predictive efficacy by ROC curve and calibration curve. Multiple variables, such as age, respiratory rate, D-dimer, LDH, and albumin, were included in the model. By combining these five variables, the model can accurately predict the occurrence of moderate COVID-19, especially for patients aged ≤66 years.
